# Barriers and facilitators of engagement with app-based pain self-management strategies among chronic pain patients (CPPs)

**DOI:** 10.1177/13591053251406436

**Published:** 2026-01-15

**Authors:** Rebecca P. Harding, Michael Passaportis, Eleanor Miles, Faith Matcham

**Affiliations:** 1 University of Sussex, Falmer, UK

**Keywords:** chronic pain, digital interventions, COM-B, behaviour change, self-management

## Abstract

This study explored barriers and facilitators to digital self-management engagement among individuals with chronic pain. Semi-structured interviews were conducted with 24 participants, guided by a 32-item schedule informed by digital health adoption literature and the Capability, Opportunity, Motivation and Behaviour (COM-B) model. Reflexive thematic analysis identified key influences on engagement, which were then mapped onto the COM-B framework to inform intervention design. Barriers were linked to physical and cognitive limitations, information access, financial constraints, self-efficacy and individual differences. Facilitators included social connection, enhanced pain awareness, autonomy and accessibility. While themes aligned with most COM-B components, no clear influences were mapped to Automatic Motivation. Findings provide nuanced insights into the behavioural and contextual factors shaping engagement with app-based interventions. By applying the COM-B model, this study offers a theoretically grounded understanding of digital self-management uptake, supporting the development of more responsive and accessible interventions for people living with chronic pain.

## Introduction

Chronic pain is a public health priority (Goldberg and McGee, 2011). It is a leading cause of disability, affecting 135 billion people globally (Fayaz et al., 2016), with European prevalence estimates ranging between 12% and 48% (Rometsch et al., 2025). Back pain alone costs the NHS £400 million annually, while arthritis is projected to reach £118.6 billion by 2027 (Versus Arthritis, 2021). Chronic pain impacts inter- and intrapersonal functioning, contributing to emotional distress, sleep issues, social isolation, job loss, depression and physical inactivity (Topcu, 2018).

Historically, chronic pain treatment has relied on opiates, prescribed to manage pain (Dorflinger et al., 2013). Opioid prescriptions in the UK have increased by 400% in the last decade, contributing to adverse outcomes such as addiction, increased comorbidities and risk of misuse and death (Alenezi et al., 2021; Gilam et al., 2020). A national agenda to reduce opioid use emerged (British Medical Association, 2025), with the National Institute for Health and Care an Excellence (NICE) recommending multidisciplinary treatment and non-pharmacological approaches for managing chronic pain (National Institute of Care and Excellence (NICE), 2021).

Non-pharmacological approaches aim to reduce pain’s negative effects, decrease the pain experience and improve self-efficacy (Geziry et al., 2018). Due to growing clinic waiting times, non-pharmacological interventions remain inaccessible to many chronic pain patients (CPPs; Deslauriers et al., 2020). Pain self-management is a core feature of non-pharmacological approaches, commonly including low-impact exercise, meditation, mindfulness and pain tracking that patients can engage in, outside of the clinical setting (Svendsen et al., 2020).

Mobile health (mHealth) expands accessibility to self-management, offering a cost-effective solution (Weatherly et al., 2024). App-based self-management interventions include education, monitoring and self-management intervention strategies (Devan et al., 2019) and support patients to develop strategies to manage their pain in the comfort of their own home (Najm et al., 2019). However, uptake and long-term use of pain applications is poor, often due to a lack of user or provider engagement and failure to incorporate behaviour change theories (Huber et al., 2017).

**Figure 1.**
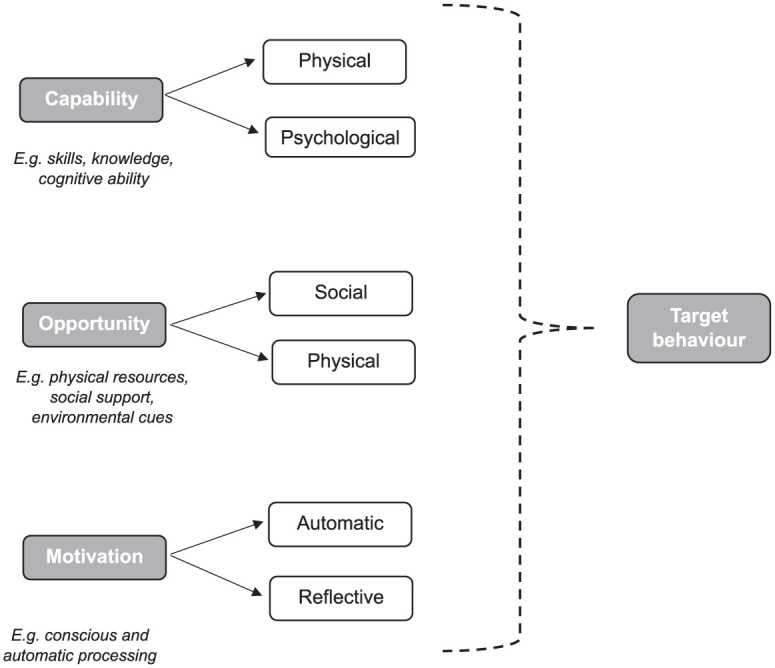
COM-B Model (Michie et al., 2011).

The Behaviour Change Wheel (BCW: (Michie et al., 2011) can guide the development of behaviour change interventions, identifying components to promote the uptake and use of interventions. It consists of three layers with the Capability, Opportunity, Motivation and Behaviour (COM-B; Michie et al., 2011; Figure 1) model at its core. It can be applied to specific behaviours or populations, highlighting factors to target for behaviour change (Ojo et al., 2019). The model outlines Capability, Opportunity and Motivation as components driving behaviour, asserting that behaviour change requires all three (Johnson et al., 2024). Capability refers to physical and psychological ability; Opportunity involves environmental factors and social influences; and Motivation encompasses the drive to engage. The COM-B model has been widely incorporated into intervention designs across various domains to facilitate behaviour change, highlighting its broad utility (McDonagh et al., 2018). It has also been applied to user engagement of mHealth applications by providing a comprehensive set of behavioural influences and theory-driven evaluation of user engagement in app-based interventions (Szinay et al., 2021).

Current understanding of engagement with digital interventions for chronic pain have not been investigated through a behaviour change lens. The aim of the current study is to investigate pain patients’ experiences with digitalised self-management interventions, map these against evidence-based COM-B behaviour change techniques to identify theoretically underpinned, service-user informed barriers and facilitators of engagement with mHealth self-management.

## Methods

### Design

We implemented a qualitative design, mapping semi-structured interview responses onto the COM-B model (Michie et al., 2011), adopting a critical realist ontological position – acknowledging both realism and the influence of participants’ unique social context and experiences (Willig, 2021) – alongside a social constructionism epistemology approach; acknowledging subjectivity and emphasising how knowledge is constructed through social interactions (Turin et al., 2024). The study was conducted and written up according to COREQ guidelines (Tong et al., 2007,Supplemental Table 1).

### Participants

We purposively sampled adults (⩾18 years) with non-cancer pain lasting over 3 months who were fluent in English. Pain status was confirmed using the Brief Pain Inventory (BPI, Cleeland, 1991). We recruited participants with and without prior experience of app-based self-management, capturing diverse perspectives on access and supporting a holistic understanding of engagement. Systematic review evidence suggests that in relatively homogenous samples, theoretical saturation tends to be achieved within 9–17 interviews (Hennink and Kaiser, 2022). As we included those with and without prior experience with app-based self-management, we planned to over recruit by approximately 30%, allowing a wider range of perspectives. Our total planned sample was 22.

### Materials

We used the BPI (Cleeland, 1991) to collect demographic details and pain characteristics and developed a 32-item interview schedule (Supplemental Table 2). This was informed by previous research pertaining to digital technology uptake (Rees et al., 2023) and the COM-B model (Michie et al., 2011).

### Procedure

Participants were recruited through an online research recruitment platform (Prolific, (https://www.prolific.com/) and word of mouth. Interested individuals met with the lead researcher (RPH) via Microsoft Teams to confirm eligibility. Interviews were conducted remotely to increase accessibility and remove organisational barriers to attending the interview. Participants provided written consent and completed baseline assessment via Qualtrics (https://www.qualtrics.com/) survey. Interviews were recorded and stored on a password-protected laptop and transcribed verbatim. Identifiable information was removed during transcription, and participants were assigned a participant number, linking them with their baseline demographic surveys. The study was approved by The University of Sussex’s ethical committee (ER/RH600/1).

### Data analysis

This research was embedded in a larger study, and analysis focused on responses from question 17 onwards (Supplemental Table 2). Data were analysed with NVivo-14, according to all six phases of Braun and Clarke’s (2021) reflexive thematic analysis (RTA): data familiarisation; initial coding; generating initial themes; theme development and review; theme refinement, finalising and naming; and writing up. RTA was selected due to its ability to identify rich patterns of meaning across larger datasets while capturing participants lived experiences (Braun and Clarke, 2021). In line with RTA principles, we acknowledged the interviewer’s active role in shaping the data and treating researcher subjectivity as a valuable analytic resource (Braun and Clarke, 2021). RPH conducted all interviews, drawing on their background in health psychology to build rapport and elicit rich accounts. RPH led coding and theme development, with all authors reviewing and refining themes through iterative, reflexive discussion to ensure the analysis remained grounded in the data while critically engaging with our own assumptions.

Phase 1 involved RPH familiarising themselves with transcripts through repeated reading and listening, noting initial points of interest (e.g. brain fog’s impact on pain self-management). In Phase 2, transcripts were coded for barriers (e.g. hopelessness, pain levels) and facilitators (e.g. social interaction, simplicity), based on interpretation of participant accounts and recognition that some factors could act as both barriers and facilitators depending on context (e.g. app simplicity). Related codes were grouped by semantic or conceptual similarity to support Phase 3. Overarching themes were generated in Phase 4, and refined and named via team discussion in Phase 5. To ensure rigour, we developed themes through collaborative reflexivity, actively discussing and challenging each other’s interpretations to deepen analysis. Coded extracts were reviewed by three critical friends (Noor and Shafee, 2021), , who questioned assumptions, offered alternative perspectives and helped resolve interpretive differences. Once themes were finalised, they were mapped onto the COM-B model in accordance with its component definitions, following a deductive approach. This mapping process was critically reviewed by FM, a chartered Health Psychologist with expertise in the BCW framework.

Participants were invited to review the findings (Lindheim, 2022) and reflect on any contradictions or alternative perspectives, constituting a final stage of data collection (Smith and McGannon, 2018). Of those who participated (6/24), all confirmed that the findings accurately reflected their experiences of barriers and facilitators to using pain-related apps, with no additional input: “I have read through the conclusions and agree whole heartedly with your findings” (P5).

## Results

**Figure 2.**
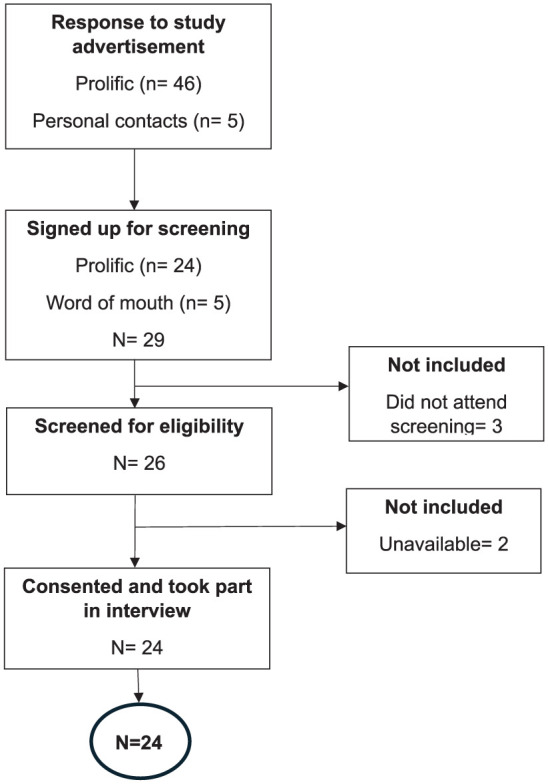
Participant recruitment flow chart.

Figure 2 shows the participant recruitment process. A total of 24 people aged 22–65 (*M* = 39.35, SD = 14.22, *F* = 20, *M* = 4) were enrolled in the study (Table 1), and interview duration ranged from 45 to 60 minutes. Additional pain characteristics are available in Supplemental Table 3.

**Table 1.** Participant demographics and pain characteristics (BPI, Cleeland, 1991).

Participant characteristic	*N*	%
Gender		
Female	20	83
Male	4	17
Employment		
Employed full time	11	46
Employed part time	3	12.5
Unemployed	2	8
Homemaker	1	4
Retired	4	17
Other	3	12.5
Pain duration		
3 months–2 years	5	21
3–5 years	7	29
6–9 years	2	8
10–19 years	4	17
20+ years	5	21
Average pain level (0–10)		
Mild (0–4)	12	50
Moderate (5–6)	9	37.5
Severe (7–10)	3	12.5

**Figure 3.**
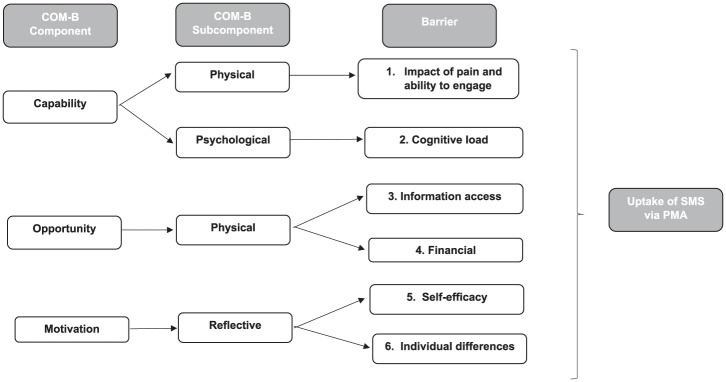
Barriers mapped onto the COM-B model.

**Figure 4.**
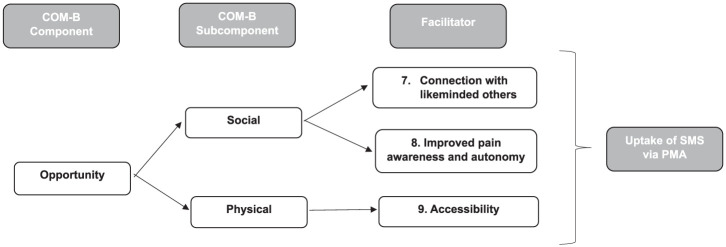
Facilitators mapped onto the COM-B model.

Nine themes were generated through RTA; six identifying barriers to engagement in self-management, and three identifying facilitators of engagement (Figures 3 and 4). Barriers included: (1) Impact of pain and ability to engage; (2) Cognitive load; (3) Information access; (4) Financial; (5) Self-efficacy and (6) Individual differences. Facilitators represented: (7) Connection with like-minded others; (8) Improved pain awareness and autonomy and (9) Accessibility. Thematised barriers and facilitators represented *Psychological* and *Physical Capability, Physical* and *Social Opportunity* and *Reflective Motivation*. Additional quotations are available in Supplemental Table 4.

### Barriers

#### Impact of pain and ability to engage (Physical capability)

Participants’ physical experience of pain and accompanying symptoms (brain fog, fatigue, and mental health difficulties) was a barrier to engagement in app-based self-management. Participants expressed how pain limited their ability to engage in self-management, acknowledging the difficulty in shifting focus to management strategies when experiencing a pain-flare up:

Well, the pain alone will stop you, cause you can’t be bothered, cause you’re too focused on your pain. (P20)

Participants paired the physical sensation of pain with the emotional response to pain as a barrier to engagement, often expressing feelings of hopelessness and doubts as to whether self-management would be effective:

I think when you’re in a lot of pain, like its hard sometimes to not think about anything else other than the fact that you are in pain. Erm and sort of just be like ‘well, nothing going to work and I’ve kind of just got to wait for it to end. (P8)

Further, accompanying symptoms of chronic pain influenced engagement in self-management. A pain-flare up was associated with reduced motivation to engage and exacerbated by common mental health difficulties faced by participants:

Sometimes you’re so miserable that you-it’s hard to engage with anything, let alone something that might help. Erm, I think chronic pain from my experience causes anxiety and depression. (P19)

#### Cognitive load (Psychological capability)

Participants discussed how common side effects of chronic pain impacted their cognitive load, and their ability to search for and engage with app-based interventions. Participants explained how they frequently forgot what activities may help manage their pain in-the-moment. This was attributed to consistently trialling new methods – resulting in cognitive overload. Thus, participants highlighted their concerns over “forgetting” to engage with digital interventions:

Erm, just because I feel like I’m trying things all the time. Erm like I said, if there wasn’t reminders, I’ll probably forget to use it. Erm, just ‘cause my mind is scattered all over the place, but that wouldn’t-that wouldn’t be a reason I would not use it – or would choose not to use. It would just be because I’ve forgotten (P18)

This barrier was also applied to lack of knowledge, with participants noting that they did not always have the energy to search for potentially effective app-based interventions:

‘Cause I don’t have the energy to be like searching everywhere and I think for me anyway, that’s the massive thing that gets in the way of just not knowing these things exist. Like not knowing that exists, not knowing these strategies exist. I don’t know how you improve that, but as far as you know, maybe social media and stuff. (P24)

#### Information access (Physical opportunity)

Participants’ lack of access to information that showcased the benefits of self-management interventions for chronic pain, and how digital applications may support pain-management was acknowledged as a barrier to engagement. Participants reported that being unaware of the availability of such applications was a primary barrier to engagement:

I think for me anyway, that’s the massive thing that gets in the way, just not knowing these things exist. (P24)

It just didn’t occur to me, and it should have, but it didn’t. (P2)

I think not hearing about them is definitely a big barrier. (P3)

#### Financial (Physical opportunity)

The potential cost of app-based support was seen as a physical barrier to engagement and uptake. Participants highlighted how most applications required payment, leading to reluctance or inability to engage:

I think the reason I only stopped using it, to be honest, was because there were certain features that I know you have to pay for and I think in that sense it’s not always financially accessible for people should you wish to use some more features. (P10)

Participants discussed how they often feel desperate for a “cure” and pay premium prices to feel let down by the results. This experience led to apprehension around paying for an application without knowledge of an evidenced benefit:

I mean there’s a financial aspect. If you had to pay for them, I think, you know people, I think just throw out a lot of money and I think people get burnt quite a lot by snake oil so to speak (P20)

Financial accessibility was also discussed within the context of chronic pain patients as a disfranchised group. Some participants reflected on their own experiences of job loss or reduced hours because of their pain, resulting in little disposable income. As such, prioritising paying for applications over basic needs was suggested to be unrealistic, especially considering the cost-of-living crisis:

I know things like apps aren’t very much money, but when you’ve got so little disposable income, the little amounts to me add up to big amounts. And you know, if-if an app a month or whatever, is two or three meals, then I’d rather eat decent food than, than pay for an app. (P5)

#### Self-efficacy (Reflective motivation)

Participants’ perceived inability to plan and perform self-management properly was seen to be a significant barrier to engagement. Participants identified their careers and busy schedules as primary obstacles of engagement. This was exacerbated by participants’ preconceived belief of self-management being time-consuming.

I’ll go home, I’ll download the app and then download it and never open it because you don’t have the time. You’re not taking the time to go on there every day (P6)

Participants demonstrated intention to engage, but a perceived inability to plan for the behaviour engagement within the constraints of their lifestyle. Participants also noted that the potential everyday commitment to engage with an application created feelings of expectation and anticipated experiencing guilt and/or shame if they were unable to engage accordingly. This anticipated emotional response was associated with disengagement and highlighted how participants’ belief that they would not be able to maintain the behaviour, acted as a barrier to initiating the behaviour. The barrier of self-efficacy was also extended to participants fear of not executing self-management properly, showcasing limited self-belief in their capabilities. Participants expressed anxiety and reluctance to engage in self-management, anticipating that they would not effectively perform the exercise:

Um, if they’re not getting it right, why should they bother doing it? Erm, you know why put in the effort to try and it’s not right? You know, why waste energy doing that, if you could be doing something else that would be more comfortable for you. You know why, why take the time and do something you’re uncomfortable with, if you could stay in your comfort zone and do something you’d prefer to do? (P6)

This fear was further compounded by the potential of performing self-management incorrectly and causing further harm to the impacted area:

So it kind of makes you feel like I just, I don’t want to do that because I don’t want to make things worse, and it’s just that fear. (P21)

#### Individual differences (Reflective motivation)

Lack of consideration of individual differences was considered as a significant barrier to engagement. Participants discussed the nuances of chronic pain presentation, stating there is no “one size fits all” approach to managing chronic pain, calling for personalisation of app-based interventions.

It’s just, because things, most of the time, the things they’re giving are quite generic. And it doesn’t take sort of everything into account. So yes, what they’re saying would probably work as a general rule, but if you’ve got something like I have, that’s got so much going on, they don’t take into account that it could aggravate other things, which then makes it worse in the long run. (P21)

Participants acknowledged how pain variability uniquely impacts their psychological state and ability to engage in app-based interventions, supporting the need for personalisation:

You know, one day you don’t, might not want some woman telling you how to do yoga and you might just want to have a bit of relaxing music. Or you might just want someone to say right, walk now, or do what you feel best or that you know, I think options and choices, yeah. (P4)

Nearly all participants expressed the desire for an application to fit their specific preferences and needs (i.e. reminder preferences and push notifications) – suggesting personalisation would overcome the barrier of individual differences and go the “extra mile”:

It would suit your needs, it would, it would be, you know, personalised to what would work for me. So, if it was, if it, if everybody all got the notifications at 5;00 o’clock how many of those people every day- how many of those people are actually gonna go and do anything? Whereas if they got it at a time that they’ve set, that they feel like you know, could be a good time for me to do it, there’s a higher chance of sort of going on and having a little look then at what’s what. (P6)

### Facilitators

#### Connection with like-minded others (Social opportunity)

Loneliness was described as a common experience for CPPs. Participants touched on the “isolating” nature of chronic pain and how this was exacerbated by the stigmatisation of chronic pain. Participants shared how they often felt misunderstood by friends, family or healthcare professionals (HCPs) and often felt like a “burden.” Further expressing the desire to connect with peers in the hopes to receive “validation” and support, as well as share ideas on how to manage their condition:

I do think it’s when someone else is suffering from chronic pain, you could, you could bounce ideas off each other and maybe talk about techniques that help you, or wallow in self-pity- whichever you like. (P4)

#### Improved pain awareness and autonomy (Physical opportunity)

Participants noted that app-based self-management could enhance their understanding of their pain presentation and environmental triggers, fostering autonomy and empowering them to make sustainable changes. This was often framed in terms of gaining insights into pain fluctuations and variability:

I struggle sometimes to notice when I’m coming into a flare up of pain and I never really have much- I don’t really put a lot of effort into tracking that. But I think if I did put more into that and something made it more accessible and easy for me to do, I might be able to then make more sustainable changes that were going to impact the overall experience. (P9)

Some participants also expressed how having greater insight of their pain may reduce their tendency to catastrophise their pain, shifting their perspective to their pain being manageable:

Because again, it’s making you think, ‘look, you’re not a seven or an eight all the time. Let’s just look at this you know, objectively’. And it makes it feel more deal-able. Sometimes your brain, is sort of just ‘ahh a horrendous night!’. You know and actually, ‘no you had three hours of really good sleep. Yes, you were 20 minutes sorting that out, 10 minutes sorting that out’- but actually, it’s not too bad. It stops the catastrophising maybe (P19)

Increased pain-insight was also viewed to increase CPPs’ autonomy. With participants often explaining how an improved understanding of their pain, would allow them to become a proactive member in their pain-management plan:

Because otherwise, I think you can just be a patient you know, a sad person who has pain or whatever. Um, whereas if you are the one who’s in control, then you are managing your pain. It’s-it’s not something that’s being inflicted on you. It’s a fact of life that you are then, dealing with. It’s it’s about, you know, taking positive steps and you know self-esteem really. (P17)

Improved pain-insights and autonomy was also viewed to increase CPPs’ ability to advocate for themselves in clinical settings. Participants felt as though tangible evidence of their symptoms and symptom frequency would help them feel less stigmatised by their chronic pain. Additionally, participants shared how a comprehensive collection of self-management, medication adherence and pain variability may support HCPs in understanding or diagnosing their pain condition. This was viewed as particularly useful considering NHS strain and reduced appointment availability:

I suppose if you had, if you were drawing on a big database of potential, potential explanations as to why you are getting your symptoms and- that might be quite useful, Uhm not, you know, there may be some situations where there is something that can be treated that, that just nobody’s aware of, or again GPs being so busy, you know, do not really have a lot of time to drill into every complaint that you’ve got. (P12)

#### Accessibility (Physical opportunity)

Participants expressed how inclusivity was a key facilitator in engaging with app-based self-management. Participants discussed the difficulty that some CPPs face using digital technology, highlighting the importance of age and disability consideration (including those who are hard of hearing and blind). Participants acknowledged how chronic pain presentation varies and commonly drew reference to those with arthritis. Participants reflected on their experiences, or their peers, of not being able to engage with digital technology because of fine text, small buttons, bright screens and lack of voice recognition. Participants shared how disability consideration and relevant adjustments would provide them, and others and increased opportunity to engage:

Sometimes it can be really hard to press it like, especially if you’ve got like arthritis in your hands and stuff like that. And so, I feel like big buttons and an easy to navigate app, because you don’t want to log on to an app and then have to do like ten other things to get what you want to do. (P7)

This notion of inclusivity was extended to older age groups and less common pain conditions. Participants acknowledged the importance of reassuring older age groups of the safety of the application (data confidentiality and storage), and ease of use. Furthermore, some participants expressed that they had previously felt underrepresented in applications – exacerbating the already prevalent feelings of isolation associated with chronic pain:

My pain and my condition wasn’t on there, and that alienated me right from- I think that basically just made me think, well it was part alienation and part again, what referring back to what I said earlier- that imposter syndrome. I think I’ve kind of, you know entered a club here that I’m not a proper member of.. (P19)

## Discussion

We identified potential barriers and facilitators to engaging in app-based self-management, among chronic pain patients.

The impact of pain and ability to engage influenced engagement in pain-based applications, highlighting how physical sensations and symptoms pose a barrier to digital self-management. This aligns with research highlighting the physical and emotional limitations of chronic pain as obstacles to sustained engagement (Bair et al., 2008). Depression, anxiety and distress further affect both the ability and the motivation to engage (Devan et al., 2018). Variability in pain and chronic pain comorbidities also hinder engagement with effective pain self-management such as low-impact physical activity (Leese et al., 2024; Schmidt et al., 2015). These findings represent *Physical Capability* – the physical strength and skills required to perform a behaviour (Michie et al., 2011) and as such demonstrate the need for interventions addressing both physical symptoms and psychological barriers to motivation and sustained participation.

The cognitive load barrier demonstrated CPPs’ limited energy and resources, affecting their ability to engage in self-management or seek strategies. For pain patients, everyday tasks like hygiene and cooking can be exhausting, leaving little energy for self-care (Arman and Hök, 2016). Consequently, basic needs often take precedence over self-management. Cognitive functions such as attention, memory and problem-solving are crucial for tasks like medication adherence and self-monitoring (Hjelm et al., 2012). Cognitive load was consistent with the COM-B construct of *Psychological Capability*: the necessary knowledge and mental skills required to perform the target behaviour. The heightened cognitive load many CPPs face can hinder engagement in self-management. This highlights the need for improved awareness of self-management applications among pain patients, and how task minimisation (i.e. reducing cognitive load to complete a task) should be prioritised in application design to increase accessibility and usability among this population.

Limited access to information was considered another key barrier to engagement. Most participants lacked awareness of digital tools for pain-management and their efficacy. With over 2 million people waiting over 18 weeks to see an HCP (British Medical Association, 2025), increasing access to low-cost interventions during this wait could reduce clinic burden and help patients build self-management skills (Hadi et al., 2017). *Physical Opportunity –* the presence and availability of relevant resources or tools to perform a behaviour – underscores the barrier of information access and acknowledges the importance of information-sharing. Thus, gathering evidence of efficacy and integrating digital solutions into NICE guidelines may encourage HCPs to recommend digital aids for symptom management, in turn increasing CPP’s physical opportunity to engage (Gavin et al., 2024).

Participants expressed dissatisfaction with subscription-based applications, consistent with research showing that cost deters engagement (Wu et al., 2017) Financial constraints, already linked to disengagement in digital interventions, may be even more significant for CPPs (Woodward et al., 2023). Chronic pain’s impact on employment and socioeconomic status compounds this issue, potentially worsening health inequalities (de Sola et al., 2016). *Physical Opportunity* was also present in this barrier, suggesting that reducing the financial cost of applications may create a greater opportunity for pain patients to engage in app-based self-management. Reducing the financial cost of applications is essential to making self-management more accessible and equitable for this population (Heinsch et al., 2022).

Self-efficacy was acknowledged as a salient barrier to engaging with self-management applications, reflecting participants’ perceived inability to plan and perform self-management. High self-efficacy is linked to better uptake, adherence and motivation, fostering positive health outcomes (Jackson et al., 2014). Among CPPs, self-efficacy improves pain-related outcomes and management (Karasawa et al., 2019), while lack of self-efficacy is a well-documented barrier (Jerant et al., 2005). Participants expressed intention to engage in self-management but doubted their ability to plan or perform self-management effectively, citing limited self-belief and movement-related fear-factors known to affect adherence (Bunzli et al., 2015). Despite these concerns, intention to engage is promising, given its link to action planning and self-efficacy (Shokrgozar and Niyari, 2024). Peer support and personalisation, both present in findings, are associated with increased self-efficacy (Bonsaksen et al., 2012; Jones, 2023). Providing platforms for home-based self-management may further boost self-efficacy and engagement, supporting the need for theoretically driven applications (Jerant et al., 2005). The barrier of Self-efficacy represents *Reflective motivation* – the conscious process of evaluating potential consequences associated with the behaviour (Michie et al., 2011). Boosting self-efficacy through providing remote support, personalisation and peer support may enhance *Reflective motivation* through increasing individuals’ confidence and willingness to engage in self-management behaviours (Liang et al., 2021).

The final barrier to engagement was Individual differences. Lack of consideration of individual differences in commercially available app-based interventions was acknowledged as a barrier to engagement by participants. Nuances of chronic pain and pain presentation demonstrates the unique needs of CPPs; supporting the notion that “one size does not fit all” (Heelas and Barker, 2021). Lack of personalisation is associated with disengagement in applications – highlighting the necessity to adopt a personalised approach in intervention design (Svendsen et al., 2020). Adopting a tailored approach supports the recommendations of the management of chronic pain as set of by NICE guidelines (NICE, 2021). Individual differences represent *Reflective Motivation* – furthering the support for a tailored approach to facilitate individual relevance of pain-management applications, enhancing the perceived value and benefit of engaging in app-based self-management (Michie et al., 2011).

Connection with like-minded others was considered as a facilitator to engagement- participants’ expressed their desires to connect with other patients. Family, friends and peers significantly influence self-management uptake and engagement (Carbone et al., 2007). Unsupportive relationships can hinder adherence to self-management by undermining confidence, discouraging behaviour change or creating practical and emotional barriers to following self-management plans (Devan et al., 2018). Conversely, peer support boosts motivation to engage in app-based self-management and improves self-efficacy, both key behavioural targets (Stenberg et al., 2022). Representing *Social Opportunity –* the social influences that can encourage or deter behaviour (Michie et al., 2011) – connection with like-minded others (i.e. peer support) could increase app-based self-management uptake while addressing the biopsychosocial needs of chronic pain management (e.g. emotional support, validation of experiences, shared coping strategies, and encouragement for physical activity; Stenberg et al., 2022).

Improved pain awareness and autonomy reflected patients’ desire to understand pain variability and triggers, enabling a more active role in self-management. Research identifies autonomy as a powerful driver of engagement, particularly in chronic pain populations (Jongen et al., 2017). Greater may awareness fosters proactive participation in management and supports behaviour maintenance, critical for chronic conditions (Hüppe et al., 2019). Proactive patients are more likely to engage in techniques like goal setting and achievement, which influence pain-related behaviours (Bair et al., 2009). Thus, fostering awareness and autonomy is essential for enhancing engagement, promoting sustained behaviour change, and improving self-management outcomes.

Finally, accessibility emphasised the need for future self-management applications to improve inclusivity (e.g. age, disability, rare conditions) to enhance engagement for patients. For example, individuals over 65, disproportionately affected by chronic pain, face unique barriers in accessing digital interventions such as scepticism about the effectiveness of digital therapies (Fayaz et al., 2016). Research highlights that older adults often question the efficacy of digital treatments, a significant barrier to uptake (Austrian et al., 2005). These doubts may be exacerbated by low digital literacy (O’Reilly et al., 2022). Improving digital literacy in older adults could increase app-based self-management uptake and reduce healthcare costs. Accessibility must also account for disabilities. Chronic pain, a highly comorbid condition, often intersects with disabilities like visual impairments, creating barriers to digital self-management and exacerbating health inequalities (Arati et al., 2015). Also representing *Physical Opportunity* (Michie et al., 2011) this theme highlights the urgent need for disability awareness in the design and implementation of digital interventions to increase the opportunity for all pain-patients to engage (Lippincot et al., 2020).

### Strengths and limitations

A strength of our study is the inclusion of participants with and without prior experience using digital pain app-based self-management. Those with experience offered insights into features affecting sustained use, while those without highlighted initial perceptions and anticipated challenges. This range of perspectives enriched our analysis and helped identify barriers and facilitators across different stages of engagement. Another strength to the study was the adoption of a theoretically driven approach to understanding the barriers and facilitators to engaging in app-based self-management among patients. As such, the findings may lay the foundation for the development of an intervention underpinned by health behaviour theory to improve engagement, adherence and positive outcomes for patients (Michie et al., 2014). Furthermore, the results showcase desires for a more personalised approach to support their pain management – showing promise of the acceptability and efficacy of a chronic pain adaptive intervention.

Recruiting through online channels assumed a level of digital literacy. This may be problematic, considering digital literacy is a well-documented barrier to engagement, specifically among the ageing population (Austrian et al., 2005). Although the higher prevalence of chronic pain in women (38%) than men (30%) aligns with broader trends (National Health Services (NHS), 2018), our sample (83% female) was overrepresented by women, potentially limiting generalisability and overlooking gender-specific self-management factors. There is also the potential for researcher bias and the complex, overlapping nature of behavioural influences, means that some barriers and facilitators could relate to multiple COM-B components. However, using multiple reviewers and confirming our analysis findings with the participants adds confidence to our interpretation of the data. We did not incorporate the perspective of HCPs who play an influential role in informing and encouraging self-management among this population (Varsi et al., 2021). Research has identified HCPs caseload and capacity as a barrier to information access for patients; thus this may be an important piece of the puzzle in enhancing and rectifying lack of knowledge (Burgess et al., 2014). Future studies should prioritise the involvement of HCPs in understanding the barriers and facilitators to engaging in digitalised self-management in this population to address current gaps in knowledge and ensure multidisciplinary involvement in intervention design and implementation (Lalloo et al., 2015).

## Conclusion

This study provides insight into perceived barriers and facilitators to engaging with app-based self-management interventions in chronic pain. Employing COM-B model mapping facilitated the identification of key behavioural components that influence uptake and utilisation of self-management via digital interventions, providing a foundation for theoretically underpinned intervention development and implementation (Michie et al., 2014). The present findings explain the importance of *Psychologica*l and *Physical Capability*, *Physical* and *Social Opportunity* and *Reflective Motivation* in decision-making around self-management and digital intervention use. As chronic pain remains a global health concern, this study shows promise to the acceptability and efficacy of a theoretically driven, tailored, digital intervention to support CPPs and reduce the psychological, physical and economic burden of chronic pain on an individual and societal level.

## Supplemental Material

sj-docx-1-hpq-10.1177_13591053251406436 – Supplemental material for Barriers and facilitators of engagement with app-based pain self-management strategies among chronic pain patients (CPPs) Supplemental material, sj-docx-1-hpq-10.1177_13591053251406436 for Barriers and facilitators of engagement with app-based pain self-management strategies among chronic pain patients (CPPs) by Rebecca P. Harding, Michael Passaportis, Eleanor Miles and Faith Matcham in Journal of Health Psychology

## References

[bibr1-13591053251406436] Alenezi A Yahyouche A Paudyal V (2021) Current status of opioid epidemic in the United Kingdom and strategies for treatment optimisation in chronic pain. International Journal of Clinical Pharmacy 43(2): 318–322. 33252724 10.1007/s11096-020-01205-y PMC8079300

[bibr2-13591053251406436] Arati K Sayali A Sushanta D , et al. (2015) Object recognition in mobile phone application for visually impaired users. Journal of Computer Engineering Epub ahead of print 2015.

[bibr3-13591053251406436] Arman M Hök J (2016) Self-care follows from compassionate care – Chronic pain patients’ experience of integrative rehabilitation. Scandinavian Journal of Caring Sciences 30(2): 374–381. 26395196 10.1111/scs.12258

[bibr4-13591053251406436] Austrian JS Kerns RD Reid MC (2005) Perceived barriers to trying self-management approaches for chronic pain in older persons. Journal of the American Geriatrics Society 53(5): 856–861. 15877564 10.1111/j.1532-5415.2005.53268.x

[bibr5-13591053251406436] Bair MJ Matthias MS Nyland KA , et al. (2009) Barriers and facilitators to chronic pain self-management: A qualitative study of primary care patients with comorbid musculoskeletal pain and depression. Pain Medicine 10(7): 1280–1290. 19818038 10.1111/j.1526-4637.2009.00707.x PMC2884223

[bibr6-13591053251406436] Bair MJ Wu J Damush TM , et al. (2008) Association of depression and anxiety alone and in combination with chronic musculoskeletal pain in primary care patients. Psychosomatic Medicine 70(8): 890–897. 18799425 10.1097/PSY.0b013e318185c510 PMC2902727

[bibr7-13591053251406436] Bonsaksen T Lerdal A Fagermoen MS (2012) Factors associated with self-efficacy in persons with chronic illness. Health and Disability 53(4): 333–339. 10.1111/j.1467-9450.2012.00959.x 22680700

[bibr8-13591053251406436] Braun V Clarke V (2021) Thematic analysis: A practical guide. Research Methods in Psychology 2.

[bibr9-13591053251406436] British Medical Association (2025) NHS backlog data analysis. Available at: https://www.bma.org.uk/advice-and-support/nhs-delivery-and-workforce/pressures/nhs-backlog-data-analysis (accessed 22 September 2025).

[bibr10-13591053251406436] Bunzli S Smith A Schütze R , et al. (2015) Beliefs underlying pain-related fear and how they evolve: A qualitative investigation in people with chronic back pain and high pain-related fear. BMJ Open 5(10): 8847. 10.1136/bmjopen-2015-008847 PMC4611881 26482773

[bibr11-13591053251406436] Burgess DJ Phelan S Workman M , et al. (2014) The effect of cognitive load and patient race on physicians’ decisions to prescribe opioids for chronic low back pain: A randomized trial. Pain Medicine 15(6): 965–974. 24506332 10.1111/pme.12378

[bibr12-13591053251406436] Carbone ET Rosal MC Torres MI , et al. (2007) Diabetes self-management: Perspectives of Latino patients and their health care providers. Patient Education and Counseling 66(2): 202–210. 17329060 10.1016/j.pec.2006.12.003

[bibr13-13591053251406436] Cleeland CS (ed.) (1991) Pain Assessment in Cancer, 1st edn. CRC Press.

[bibr14-13591053251406436] Deslauriers S Roy JS Bernatsky S , et al. (2020) The association between waiting time and multidisciplinary pain treatment outcomes in patients with rheumatic conditions. BMC Rheumatology 4(1): 12. 10.1186/s41927-020-00157-0 PMC7583241 33111034

[bibr15-13591053251406436] de Sola H Salazar A Dueñas M , et al. (2016) Nationwide cross-sectional study of the impact of chronic pain on an individual’s employment: Relationship with the family and the social support. BMJ Open 6: e012246. 10.1136/bmjopen-2016-012246 PMC5223634 28011806

[bibr16-13591053251406436] Devan H Hale L Hempel D , et al. (2018) What works and does not work in a self-management intervention for people with chronic pain? Qualitative systematic review and meta-synthesis. Physical Therapy 98(5): 381–397. 29669089 10.1093/ptj/pzy029

[bibr17-13591053251406436] Devan H Perry MA van Hattem A , et al. (2019) Do pain management websites foster self-management support for people with persistent pain? A scoping review. Patient Education and Counseling 102: 1590–1601. 30981410 10.1016/j.pec.2019.04.009

[bibr18-13591053251406436] Dorflinger L Kerns RD Auerbach SM (2013) Providers’ roles in enhancing patients’ adherence to pain self management. Translational Behavioral Medicine 3(1): 39–46. 24073159 10.1007/s13142-012-0158-z PMC3717997

[bibr19-13591053251406436] Fayaz A Croft P Langford RM , et al. (2016) Prevalence of chronic pain in the UK: A systematic review and meta-analysis of population studies. BMJ Open 6(6): e010364. 10.1136/bmjopen-2015-010364 PMC4932255 27324708

[bibr20-13591053251406436] Gavin JP Clarkson P Muckelt PE , et al. (2024) Healthcare professional and commissioners’ perspectives on the factors facilitating and hindering the implementation of digital tools for self-management of long-term conditions within UK healthcare pathways. PLoS One 19(8): e0307493. 10.1371/journal.pone.0307493 PMC11343405 39178238

[bibr21-13591053251406436] Geziry AE Toble Y Al Kadhi F , et al. (2018) Non-pharmacological pain management. In: Shallik NA (ed.) Pain Management in Special Circumstances. IntechOpen, pp.1–14.

[bibr22-13591053251406436] Gilam G Sturgeon JA You DS , et al. (2020) Negative affect–related factors have the strongest association with prescription opioid misuse in a cross-sectional cohort of patients with chronic pain. Pain Medicine 21(2): e127–e138. 10.1093/pm/pnz249 PMC7049262 31617916

[bibr23-13591053251406436] Goldberg DS McGee SJ (2011) Pain as a global public health priority. BMC Public Health 11: 770. 21978149 10.1186/1471-2458-11-770 PMC3201926

[bibr24-13591053251406436] Hadi MA Alldred DP Briggs M , et al. (2017) ‘Treated as a number, not treated as a person’: A qualitative exploration of the perceived barriers to effective pain management of patients with chronic pain. BMJ Open 7(6): e016454. 10.1136/bmjopen-2017-016454 PMC5541634 28606909

[bibr25-13591053251406436] Heelas L Barker KL (2021) One size does not fit all: Enabling choice and patient-centred care in a chronic pain rehabilitation service. Physiotherapy 113: e184.

[bibr26-13591053251406436] Heinsch M Tickner C Kay-Lambkin F (2022) Placing equity at the heart of eHealth implementation: A qualitative pilot study. International Journal for Equity in Health 21(1): 38. 35303883 10.1186/s12939-022-01640-5 PMC8931179

[bibr27-13591053251406436] Hennink M Kaiser BN (2022) Sample sizes for saturation in qualitative research: A systematic review of empirical tests. Social Science & Medicine 292: 292. 10.1016/j.socscimed.2021.114523 34785096

[bibr28-13591053251406436] Hjelm C Dahl A Broström A , et al. (2012) The influence of heart failure on longitudinal changes in cognition among individuals 80 years of age and older. Journal of Clinical Nursing 21(7-8): 994–1003. 21883570 10.1111/j.1365-2702.2011.03817.x

[bibr29-13591053251406436] Huber S Priebe JA Baumann KM , et al. (2017) Treatment of low back pain with a digital multidisciplinary pain treatment app: Short-term results. JMIR Rehabilitation and Assistive Technologies 4(2): e9032. 10.2196/rehab.9032 PMC5735251 29203460

[bibr30-13591053251406436] Hüppe A Zeuner C Karstens S , et al. (2019) Feasibility and long-term efficacy of a proactive health program in the treatment of chronic back pain: A randomized controlled trial. BMC Health Services Research 19(1): 714. 31639016 10.1186/s12913-019-4561-8 PMC6805578

[bibr31-13591053251406436] Jackson T Wang Y Wang Y , et al. (2014) Self-efficacy and chronic pain outcomes: A meta-analytic review. Journal of Pain 15(8): 800–814. 24878675 10.1016/j.jpain.2014.05.002

[bibr32-13591053251406436] Jerant AF von Friederichs-Fitzwater MM Moore M (2005) Patients’ perceived barriers to active self-management of chronic conditions. Patient Education and Counseling 57(3): 300–307. 15893212 10.1016/j.pec.2004.08.004

[bibr33-13591053251406436] Johnson JL Blefari C Marotti S (2024) Application of the COM-B model to explore barriers and facilitators to participation in research by hospital pharmacists and pharmacy technicians: A cross-sectional mixed-methods survey. Research in Social and Administrative Pharmacy 20: 2725–2738. 10.1016/j.sapharm.2023.10.002 37813706

[bibr34-13591053251406436] Jones T (2023) Impact of Self-Management Education on the Self-Efficacy of People with Chronic Pain. Arkansas State University, Jonesboro. Available at: https://arch.astate.edu/cgi/viewcontent.cgi?article=1064&context=dnp-projects.

[bibr35-13591053251406436] Jongen PJ Ruimschotel R Museler-Kreijns Y , et al. (2017) Improved health-related quality of life, participation, and autonomy in patients with treatment-resistant chronic pain after an intensive social cognitive intervention with the participation of support partners. Journal of Pain Research 10: 2725–2738. 29238216 10.2147/JPR.S137609 PMC5716312

[bibr36-13591053251406436] Karasawa Y Yamada K Iseki M , et al. (2019) Association between change in self-efficacy and reduction in disability among patients with chronic pain. PLOS ONE 14(4): e0215404. 10.1371/journal.pone.0215404 PMC6467389 30990842

[bibr37-13591053251406436] Lalloo C Jibb L Rivera J , et al. (2015) “There’sa pain app for that”: review of patient-targeted smartphone applications for pain management. Clinical Journal of Pain Epub ahead of print 2015. 10.1097/AJP.0000000000000171 25370138

[bibr38-13591053251406436] Leese C Gupte D Christogianni A , et al. (2024) Barriers and facilitators for physical activity in people living with chronic pain: A systematic review and combined analysis. Pain 165(12): 2721–2732. 38981051 10.1097/j.pain.0000000000003314

[bibr39-13591053251406436] Liang D Jia R Zhou X , et al. (2021) The effectiveness of peer support on self-efficacy and self-management in people with type 2 diabetes: A meta-analysis. Patient Education and Counseling 104(4): 760–769. 33229189 10.1016/j.pec.2020.11.011

[bibr40-13591053251406436] Lindheim T (2022) Participant Validation: A Strategy to Strengthen the Trustworthiness of Your Study and Address Ethical Concerns. Methodological approaches for understanding values work in organisations and leadership.

[bibr41-13591053251406436] Lippincot B Thompson N Morris J , et al. (2020) Survey of user needs: Mobile apps for mHealth and people with disabilities. In: International conference on computers helping people with special needs, pp.266–273. Springer Science and Business Media Deutschland GmbH.

[bibr42-13591053251406436] McDonagh LK Saunders JM Cassell J , et al. (2018) Application of the COM-B model to barriers and facilitators to chlamydia testing in general practice for young people and primary care practitioners: A systematic review. Implementation Science 13(1): 130. 10.1186/s13012-018-0821-y PMC6196559 30348165

[bibr43-13591053251406436] Michie S Atkins L West R (2014) The Behaviour Change Wheel: A Guide to Designing Interventions. Silverback Publishing.

[bibr44-13591053251406436] Michie S van Stralen MM West R (2011) The behaviour change wheel: A new method for characterising and designing behaviour change interventions. Implementation science : IS 6(1): 42. 21513547 10.1186/1748-5908-6-42 PMC3096582

[bibr45-13591053251406436] Najm A Gossec L Weill C , et al. (2019) Mobile health apps for self-management of rheumatic and musculoskeletal diseases: Systematic literature review. JMIR mhealth and uhealth 7(11): e14730. 10.2196/14730 PMC6904900 31769758

[bibr46-13591053251406436] National Health Services (NHS) (2018) Health Survey for England 2017 [NS]. NHS England Digital. Available at: https://digital.nhs.uk/data-and-information/publications/statistical/health-survey-for-england/2017 (accessed 21 October 2025).

[bibr47-13591053251406436] National Institute of Care and Excellence (2021) Overview | Chronic Pain (primary and Secondary) in Over 16s: Assessment of All Chronic Pain and Management of Chronic Primary Pain | Guidance | NICE. Available at: https://www.nice.org.uk/guidance/ng193 (accessed 22 September 2025). 33939353

[bibr48-13591053251406436] Noor MSAM Shafee A (2021) The role of critical friends in action research: A framework for design and implementation. Practitioner Research 3: 1–33.

[bibr49-13591053251406436] Ojo SO Bailey DP Hewson DJ , et al. (2019) Perceived barriers and facilitators to breaking up sitting time among desk-based office workers: A qualitative investigation using the TDF and COM-B. International Journal of Environmental Research and Public Health 16(16): 2903. 10.3390/ijerph16162903 PMC6720704 31416112

[bibr50-13591053251406436] O’Reilly PM Harney OM Hogan MJ , et al. (2022) Chronic pain self-management in middle-aged and older adults: A collective intelligence approach to identifying barriers and user needs in eHealth interventions. Digital Health 8: 1–15. 10.1177/20552076221105484 PMC9185015 35694121

[bibr51-13591053251406436] Rees J Liu W Ourselin S , et al. (2023) Understanding the psychological experiences of loneliness in later life: qualitative protocol to inform technology development. BMJ Open 13(6): e072420. 10.1136/bmjopen-2023-072420 PMC10314543 37336536

[bibr52-13591053251406436] Rometsch C Martin A Junne F , et al. (2025) Chronic pain in European adult populations: a systematic review of prevalence and associated clinical features. Pain 166(4): 719–73 10.1097/j.pain.0000000000003406 PMC11921450 40101218

[bibr53-13591053251406436] Schmidt A Corcoran K Grahame R , et al. (2015) How do people with chronically painful joint hypermobility syndrome make decisions about activity? British Journal of Pain 9(3): 157–166. 26516572 10.1177/2049463714554112 PMC4616977

[bibr54-13591053251406436] Shokrgozar A Niyari M (2024) Predicting treatment plan adherence based on mindfulness and self-efficacy beliefs in patients with chronic pain. Thrita 12(2): e146427.

[bibr55-13591053251406436] Smith B McGannon KR (2018) Developing rigor in qualitative research: Problems and opportunities within sport and exercise psychology. International Review of Sport and Exercise Psychology 11(1): 101–121.

[bibr56-13591053251406436] Stenberg N Gillison F Rodham K (2022) How do peer support interventions for the self-management of chronic pain, support basic psychological needs? A systematic review and framework synthesis using self-determination theory. Patient Education and Counseling 105(11): 3225–3234. 35985906 10.1016/j.pec.2022.07.017

[bibr57-13591053251406436] Svendsen MJ Wood KW Kyle J , et al. (2020) Barriers and facilitators to patient uptake and utilisation of digital interventions for the self-management of low back pain: A systematic review of qualitative studies. BMJ Open 10(12): e038800. 10.1136/bmjopen-2020-038800 PMC7735096 33310794

[bibr58-13591053251406436] Szinay D Perski O Jones A , et al. (2021) Perceptions of factors influencing engagement with health and well-being apps in the United Kingdom: Qualitative Interview Study. JMIR mhealth and uhealth 9: e29098. 10.2196/29098 PMC8726027 34927597

[bibr59-13591053251406436] Tong A Sainsbury P Craig J (2007) Consolidated criteria for reporting qualitative research (COREQ): A 32-item checklist for interviews and focus groups. International Journal for Quality in Health Care 19(6): 349–357. 17872937 10.1093/intqhc/mzm042

[bibr60-13591053251406436] Topcu SY (2018) Relations among pain, pain beliefs, and psychological well-being in patients with chronic pain. Pain Management Nursing 19: 637–644. 30181033 10.1016/j.pmn.2018.07.007

[bibr61-13591053251406436] Turin TC Raihan M Chowdhury N (2024) Paradigms of approaches to research. Bangabandhu Sheikh Mujib Medical University Journal 17(2): e73973.

[bibr62-13591053251406436] Varsi C Ledel Solem IK Eide H , et al. (2021) Health care providers’ experiences of pain management and attitudes towards digitally supported self-management interventions for chronic pain: A qualitative study. BMC Health Services Research 21(1): 778. 10.1186/s12913-021-06278-7 PMC7992849 33766028

[bibr63-13591053251406436] Versus Arthritis (2021) Unseen, Unequal and Unfair: Chronic Pain in England. Arthritis UK. Available at: https://www.arthritis-uk.org/news/2021/june/unseen-unequal-and-unfair-chronic-pain-in-england/ (accessed 21 October 2025).

[bibr64-13591053251406436] Weatherly S McKenna T Wahba S , et al. (2024) Effectiveness of digital health interventions (DHI) in chronic pain management: A scoping review of current evidence and emerging trends. Cureus 16(10): e72562. 10.7759/cureus.72562 PMC11602419 39610577

[bibr65-13591053251406436] Willig C (ed.) (2021) Introducing Qualitative Research in Psychology, 3rd edn. McGraw-Hill Education (Open University Press).

[bibr66-13591053251406436] Woodward A Davies N Walters K , et al. (2023) Self-management of multiple long-term conditions: A systematic review of the barriers and facilitators amongst people experiencing socioeconomic deprivation. PLoS One 18(2): e0282036. 10.1371/journal.pone.0282036 PMC9942951 36809286

[bibr67-13591053251406436] Wu K Vassileva J Zhao Y (2017) Understanding users’ intention to switch personal cloud storage services: Evidence from the Chinese market. Computers in Human Behavior 68: 300–314.

