# Supplementary material for: Barriers and facilitators of engagement with app-based pain self-management strategies among chronic pain patients (CPPs)
Source: J Health Psychol. 2026 Jan 15;31(9):3727–43. doi: 10.1177/13591053251406436 (PMC13365317; doi:10.1177/13591053251406436)
Supplement: sj-docx-1-hpq-10.1177_13591053251406436 – Supplemental material for Barriers and facilitators of engagement with app-based pain self-management strategies among chronic pain patients (CPPs) [file sj-docx-1-hpq-10.1177_13591053251406436.docx]

Supplementary material

**Table S1**: *COREQ checklist*

Consolidated criteria for reporting qualitative studies (COREQ): 32-item checklist

Developed from:

Tong A, Sainsbury P, Craig J. Consolidated criteria for reporting qualitative research (COREQ): a 32-item checklist for interviews and focus groups. International Journal for Quality in Health Care. 2007. Volume 19, Number 6: pp. 349 – 357

| **Item No** | **Guide Questions/Description** | **Reported on Page #** |  |  |
| --- | --- | --- | --- | --- |
| **Domain 1: Research team and reflexivity** | | |  |  |
| **Personal Characteristics** | | |  |  |
| 1. Interviewer/ facilitator | Which author/s conducted the interview or focus group? | Pg 5 |  |  |
| 2. Credentials | What were the researcher’s credentials? E.g., PhD, MD | Pg 5 |  |  |
| 3. Occupation | What was their occupation at the time of the study? | Pg 5 |  |  |
| 4. Gender | Was the researcher male or female? | Pg 28 (Supplementary 4) |  |  |
| 5. Experience and training | What experience or training did the researcher have? | Pg 28 (Supplementary 4) |  |  |
| **Relationship with participants** | | |  |  |
| 6. Relationship established | Was a relationship established prior to study commencement? | Pg 5 |  |  |
| 7. Participant knowledge of the interviewer | What did the participants know about the researcher? e.g. personal goals, reasons for doing the research? | Pg 5 |  |  |
| 8. Interviewer characteristics | What characteristics were reported about the interviewer/facilitator? e.g. Bias, assumptions, reasons and interests in the research topic | Pg 28 (Supplementary 4) |  |  |
| **Domain 2: study design** | | |  |  |
| **Theoretical framework** | | |  |  |
| 9. Methodological orientation and Theory | What methodological orientation was stated to underpin the study? e.g. grounded theory, discourse analysis, ethnography, phenomenology, content analysis | Pg 4 |  |  |
| **Participant selection** | | |  |  |
| 10. Sampling | How were participants selected? e.g., purposive, convenience, consecutive, snowball | Pg 4 |  |  |
| low 11. Method of approach | How were participants approached? e.g., face-to-face, telephone, mail, email | Pg 4 |  |  |
| 12. Sample size | How many participants were in the study? | Pg 8 |  |  |
| 13. Non-participation Setting | How many people refused to participate or dropped out? Reasons? | Pg 2 (figures and tables) |  |  |
| 14. Setting of data collection | Where was the data collected? e.g., home, clinic, workplace | Pg 5 |  |  |
| 15. Presence of nonparticipants | Was anyone else present besides the participants and researchers? | N/A |  |  |
| 16. Description of sample | What are the important characteristics of the sample? e.g. demographic data, date | Pg 3 (figures and tables) |  |  |
| **Data collection** | | |  |  |
| 17. Interview guide | Were questions, prompts, and guides provided by the authors? Was it pilot tested? | Pg 4, 28 (Supplementary table 2 and supplementary material 4) |  |  |
| 18. Repeat interviews | Were repeat interviews carried out? If yes, how many? | N/A |  |  |
| 19. Audio/visual recording | Did the research use audio or visual recording to collect the data? | Pg.5 |  |  |
| 20. Field notes | Were field notes made during and/or after the interview or focus group? | Pg.28 (Supplementary material 4) |  |  |
| 21. Duration | What was the duration of the interviews or focus group? | Pg 8 |  |  |
| 22. Data saturation | Was data saturation discussed? | Pg 4 |  |  |
| 23. Transcripts returned | Were transcripts returned to participants for comment and/or correction? | N/A |  |  |
| **Domain 3: analysis and findings** | | |  |  |
| **Data analysis** | | |  |  |
| 24. Number of data coders | How many data coders coded the data? | Pg 6, Pg 7 |  |  |
| 25. Description of the coding tree | Did the authors provide a description of the coding tree? | N/A |  |  |
| 26. Derivation of themes | Were themes identified in advance or derived from the data? | Pg 7 |  |  |
| 27. Software | What software, if applicable, was used to manage the data? | Pg 6 |  |  |
| 28. Participant checking | Did participants provide feedback on the findings? | Pg 7 |  |  |
| **Reporting** | | |  |  |
| 29. Quotations presented | Were participant quotations presented to illustrate the themes/findings? Was each quotation identified? e.g., participant number | Pg 8-17, Pg 8 (Supplementary table 3) |  |  |
| 30. Data and findings consistent | Was there consistency between the data presented and the findings? | Pg 8-17 |  |  |
| 31. Clarity of major themes | Were major themes clearly presented in the findings? | Pg 8-17 |  |  |
| 32. Clarity of minor themes | Is there a description of diverse cases or a discussion of minor themes? | Pg 6 |  |  |

**Table S2**. *Interview Schedule*

|  | **Interview questions** |
| --- | --- |
| 1 | Please describe the pain you experience.  *1a. What type of pain do you have? Is there a specific cause?* |
| 2 | How frequently do you experience chronic pain?  *2a. How long does your pain usually last?* |
| 3 | What symptoms, if any, accompany your pain? |
| 4 | How would you rate the severity of your pain on a day-to-day basis? |
| 5 | Thinking about the last time you experienced severe pain, can you remember anything happening in the days or weeks leading up to it, which might have contributed to it?  *5a. Have you had any changes to your usual routine, life events, or diagnoses?* |
| 6 | You’ve mentioned that [insert answer to Q5] happened shortly before you experienced a pain-flare up.. how long, roughly, before you started feeling  pain, did you start to experience this? |
| 7 | Is there anything that could have stopped [insert answer to Q5] from influencing your pain? |
| 8 | Is there anything else you haven’t mentioned so far that could have been associated with the onset of your most recent pain-flare up?  8a. *Feel free to mention anything, no matter how big or small it might have seemed at the time.* |
| 9 | When you think about your most recent pain flare up, how do you feel that your pain impacted your daily routine?   9a. *Describe anything that comes to mind – your mental health, social life, relationships, physical health…* |
| 10 | You mentioned that [insert answer to Q8] was associated with the pain flare up that you experienced. Can you tell me more about that?  *10a. Did they occur at the same time?*  *10b.Can you describe the experience in more detail?*  *10c.Did X finish when the pain flare up had eased and/or ended?* |
| 11 | Thinking about your most recent pain flare up, can you tell me more about the aspects of your life it affected?  11a. *Describe anything that comes to mind – your mental health, social life, relationships, physical health…* |
| 12 | How did the pain ease and/or end? Did it finish naturally, or was there an event or strategy  which helped reduce the pain? |
| 13 | If something specific helped it go away, can you explain what happened?  13a. *Did you actively seek help?*  *13b. Who did you speak to? What did they do?*  *13c. If you didn’t actively seek help, what triggered the change in your pain level?* |
| 14 | What do you think would help minimise your pain on a daily basis? |
| 16 | Is there anything else about your experience of chronic pain which you would like to share before we move onto the next part of the interview? |
| 17 | Do you practice any SMS  to manage your pain and accompanying symptoms? *This could be an app on your phone, a website you access regularly, or some kid of technology that you wear or carry with you to measure something.* |
| 18 | [If yes], please tell me more about it. How often do you do it? What’s your favourite thing about it and what’s your least favourite thing about it? |
| 19 | [If no], please tell me more about it. Is there any particular reason as to why you don’t practise SMS? Would you want to practice SMS? Is there anything that would increase the chance of you engaging? |
| 20 | What do you think might stop people from engaging in SMS? |
| 21 | What pain management applications are you familiar with? |
| 22 | Do you use any application to practice?  *22a. This could be anything you do on your own, or a guided activity on an app on your phone, a website you access regularly etc.* |
| 23 | [If yes], please tell me more about it. How often do you do it? What’s your favourite thing about it, and what’s your least favourite thing about it? |
| 24 | [If no], please tell me more about it. Why do you think you don’t use them? What do you think would make you more likely to use them in the future? What do you think could make pain related apps more appealing for yourself and others?  *24a. Describe anything that comes to mind – your overall attitude towards applications, whether you like or dislike them, whether you think they can be effective etc.* |
| 25 | What do you think might stop people experiencing chronic pain from engaging in SMS through applications? |
| 26 | If we were to ask you to use an app every day to engage in SMS, what would prevent you, or make you less willing to engage in digital SMS every day? |
| 27 | What would encourage you, or make you more likely to use an app to practice SMS every day? |
| 28 | What type of function do you any think an application should include? |
| 29 | Thinking about the conversation we’ve just had about your preferences and recommendations of for pain management applications, what might change about your opinion or requirements would change if you were experiencing a pain flare up? |
| 30 | Conversely, if you’re currently experiencing a pain flare up, What might change about your  opinions or requirements if you weren’t experiencing a pain flare up? |
| 31 | Is there anything else about your preferences or requirements for a device which you haven’t had an opportunity to mention so far? |
|  |  |

|  |  |
| --- | --- |

|  |  | N | % |
| --- | --- | --- | --- |
| Pain severity |  |  |  |
|  | Considered bearable | 9 | 37.5% |
|  | Considered unbearable | 15 | 62.5% |
| Pain and Mood interference |  |  |  |
|  | Mild interference (0-4) | 8 | 33.3% |
|  | Moderate interference (5-6) | 4 | 16.7% |
|  | Severe interference (7-10) | 12 | 50% |
| Reported management techniques |  |  |  |
|  | Warm compress | 5 | 22.7% |
|  | Cold compress | 5 | 22.7% |
|  | Distraction | 9 | 41% |
|  | Relaxation techniques | 3 | 13.6% |
|  |  |  |  |

**Table S3.**  *Participant pain characteristics (BPI, Cleeland, 1991)*

**Table S4**. *Sample quotes of barriers and facilitators mapped onto the COM-B model*

| **Barriers** |  |  |  |  |
| --- | --- | --- | --- | --- |
| COM-B component | COM-B subcomponent | Inductive theme | **Definition** | Sample quote(s) |
| Capability | Physical | 1. Impact of pain and ability to engage | This theme refers to how the physical sensation of pain, along with any accompanying symptoms, can directly affect an individual's capacity to engage in self-management via applications. Pain and other related symptoms may hinder the physical capability required to interact with or use these digital tools effectively. | It's probably the pain ironically, would probably stop it. I can't really think of anything just other than, when I have a really bad pain, sometimes I just don't want to do anything. I don't wanna, I don’t wanna even be trying to help myself when I'm having a really bad day. Which is a problem (P15)  I think it would be difficult to engage with an app if you were in a lot of pain, I think it would be difficult (P2)  Maybe its not the right time in their life for it, maybe. I don’t know, especially if the sort of bad mental health I don’t know (P6)  I think like the tiredness or like the fatigue or whatever that goes alongside, that can be a big barrier. (P9)  Uh ‘cause when you're in pain, you really don't want to engage in anything. (P4) |
| Capability | Psychological | 1. Cognitive load | This theme refers to the mental effort required to engage with self-management applications. It highlights how factors such as memory difficulties, attention, and mental fatigue can impair an individual's ability to consistently use the app as intended. As a result, these cognitive challenges reduce the effectiveness of the application for managing pain. | And for me, quite often, my memory being so bad I forget what I'm supposed to be doing. So by the time I get to remember, that I'm supposed to be looking at this app, to do something. Kind of, it might have left, I might have left it a bit long (P15)  It just seems a lot of, it would have been a lot of effort. And you’re not feeling well, and I don’t know how you would make that easier, but some days it was just like, I just like wouldn’t have the energy (P14)  I know there must be ways to improve my life and the pain levels and stuff. And I know its out there, I just can’t get it (P24)  I get to the point where when I'm on the, my husband has to remind me to eat some days because I just forget. I'll sit here and I won't eat because I won't feel great. (P7)  Having the energy and being bothered to go out of your way and do it and sort of trying to get it sort of, to fit within your routine. (P6)  Erm, I think the pain takes over your concentration. (P20) |
| Physical | Opportunity | 1. Information access | This theme refers to the availability and accessibility of information needed to effectively engage with pain self-management applications. It includes the lack of familiarity with digital tools and insufficient knowledge about which strategies will be most effective for managing pain. Limited access to information can hinder an individual's physical opportunity to use these applications and implement appropriate self-management techniques. | I’m not familiar with anything, I haven’t really, I haven’t really- like digital applications- none of them, I haven’t really explored them (P13)  Not quite knowing what strategies are gonna work best. I think lack of knowledge for what strategies will work best (P15)  I don’t think I’ve ever thought about an app for the pain only (P22)  I think mainly like, just not, its not knowing about them (P24)  I didn’t know they existed (P5)  Its never really something I’ve- I’ve thought about (P9) |
| Physical | Opportunity | 1. Financial | This theme refers to the financial barriers that can prevent individuals from accessing and engaging with pain self-management applications. For many chronic pain patients, financial strain—often caused by job loss, reduced working hours, or disability—can make it difficult to afford apps, especially those with subscription fees or additional charges for premium features. These financial barriers limit their physical opportunity to use these digital tools for pain management. | Erm if its free or not, I’m not paying- sorry (P6)  And all of, all of them wanted me to pay them, to sort of get access to more and I couldn’t justify that, because they didn’t seem to be geared up towards me (P14)  Its nothing worse than sign up and that, and then its like well, if you want this app and then you want this but with no adverts in it, or you want this, you’ve gotta pay extra. I just want it to be straightforward. If you’re gonna use this app, this is how much its gonna cost ya (P15)  I used Headspace for a while, until I think they made that costly or something happened to do with cost. So I left that, let that off my card (P19)  In the UK there’s like three and you have to pay for them- they are pants (P20)  I personally don’t want to spend any money on something, a lot of these things because very subscription based and you can be tied into these things. Erm, and not everyone, not all these kind of apps are very clear either, and they can fool a lot of people. And I also think if you’re offered a miracle cure, a lot of people hand over money and its not, you know, its not very fair (P4)  I think it’s a bit annoying that the chronic pain stuff is behind a paywall (P24)  Its financial as well. I mean, you just cant afford all the stuff really to the potential stuff (P20)  And I had to sort of like put it together myself because I- I couldn’t afford it, if I’m honest, I wont be able to afford to pay for all the different apps because I couldn’t work. So you know, it was limited, a limited outgoing that I couldn’t justify (P14)  What else do I like? Not being charged a lot (P20) |
| Reflective | Motivation | 1. Self-efficacy | \|  \| \| --- \|  \| This theme refers to the individual’s belief in their ability to successfully manage their pain through self-management applications. Fears and doubts, such as concerns about worsening pain or not performing the activities correctly, can undermine self-efficacy. For chronic pain patients, a lack of confidence in their abilities can prevent them from engaging with these tools effectively, as they may fear failure or negative outcomes. \| \| --- \| | I think worry to be honest. I think the worry that maybe it won’t work or that it could make their pain worse at some points. I know for a long period of time when I was first diagnosed, I got so scared to do any activities, that most of the time I just stayed in bed and I missed a lot of university as well as a result of it because I was so nervous about the fact that, I just wouldn’t be able to participate in the thing that other people were doing (P10)  It’s the fear of making it worse. So it kind of makes you feel like I just, I don’t want to do that because I don’t want to make things worse, and it’s just that fear (P21)  Its like you’re sat there gong ‘well, I tried to think that I’m real and like, I’m here, but I’m not sure I did it right’. And again, nobody can ever tell you- ‘yes you did that right. No you did that wrong’- because its your experience of being present right? (P11)  Uh feel like you’re not doing it properly. Erm, not feeling comfortable doing it, and that can make you feel quite anxious. (P6)  Don’t push on this, because this will you know, like break you apart if you, if you- if you’re not careful (P16)  Say you had to do it every day, and maybe I miss a day or two, there’s no point me doing it ever again, because I’ve already missed those two days. So what’s the point? And then I won’t just do it, I just dump it (P6)  I think also as well, if you are maybe, you know you have an intense career, and sort of working, sorting of frantically a nine to five, then you’re not going to have the time, even if there’s a reminder, Saying that, if you have chronic pain then its not likely you’re going to be working (P18)  I think for example if I had to do it everyday, if I was told by my therapist that I had to do something every single day, I would find a lot of pressure through that, especially if I didn’t meet the expectations. And I’d feel quite guilty or that I wasn’t hitting the supposed targets that I could be to manage my chronic pain so (P10)  Obviously for me, um it had an impact on my job at one point, and still does. Its like that, ‘well if I’m loosing income because I can’t go to work, or because I’ve got to go to appoints, you know why am I paying £20 every month for this?’ You know what I mean? Because it’s just, its money out your account and obviously with the economy at the moment (P20) |
| Reflective | Motivation | 1. Individual differences | This theme acknowledges that individual preferences, experiences, and personal circumstances can influence how people engage with pain self-management applications. Chronic pain presents differently across individuals, and many apps fail to provide the level of personalization needed to address these unique needs. Factors such as the type of pain, past experiences, and the perceived fit of the app to an individual's needs can all impact their motivation to use the tool. | We as individuals have personal preferences and it could just be, that’s not the right fit with you as a person (P18)  So, it’s a big more targeted towards me, makes me feel like it’s going the extra mile (P20)  I guess that would depend on the individual- that would depend on the person and what they’ve gone through and the pain they’ve got (P21)  Its difficult, isn’t it? Because there’s so much variation in what people have with chronic pain, isn’t It? (P12)  I’d rather do it in my own sort of time when I’m flexible to do so (P6)  Maybe it doesn’t have to be daily, maybe it’s, I don’t know, a few days a week if you want to start slow or start fast- whichever. Maybe people could have their choice of notifications, so they know how to deal with it (P4)  There’s never going to be one size fits all, because you know one day you don’t- might not want some women telling you how to do yoga, and you might just want to have a bit of relaxing music. Or you might just want someone to say ‘right, walk now, or do what you feel best or that you know’. I think options and choices yeah (P4)  I guess so just a but more like specific case, rather than quite vague guidelines would be helpful (P9)  Its just, because things, most of the time, the things they’re giving are quite generic. And it doesn’t take sort of everything into account. So yes, what they’re saying would probably work as a general rule, but if you’ve got something like I have, that’s got so much going on, they don’t tale into account that it could aggravate other things, which then makes it worse in the long run (P21)  I also think its quite hard because chronic pain, it’s so different for everyone (P24)  I think there’s too many, everyone’s very complicated, aren’t they? There’s too- there’s a lot of different type of pain so, there’s no generic thing, really. I don’t think, I’ve been, I don’t know how they’re generically going to do it really (P4)  Because I’m going back into something and I don’t need to keep setting it up or I don’t need to be thinking; oh, is this right for me?’ because I’ve already done it once. Once I’ve done it once and its personalised, I don’t have to keep thinking, ‘am I doing this right? Or is this good for someone with my condition?’ (P15)  I guess that would depend on the individual- that would depend on the person and what they’ve gone through and the pain they’ve got (P21)  I understand that, but its just, its difficult to find people who will listen and fully grasp what help you need- and I get that- but it just results in generic information given to you (P21) |
| **Facilitators** |  |  |  |  |
| COM-B component | COM-B subcomponent | **Inductive theme** | **Definition** | **Sample quote(s)** |
| Opportunity | Social | 1. Connection with likeminded others | This theme refers to the value of social connections with others who share similar experiences. Feeling understood and supported by others facing similar challenges can enhance motivation and provide a sense of solidarity. When individuals are able to connect with others who understand their pain, it can reduce feelings of isolation and increase their opportunity to engage with self-management strategies through digital tools. | I really think if you can, having someone with you to go through that experience with and really have that journey with could be helpful (P10)  Its more helpful in the sense that I feel that I’m not the only one in pain. You know, it is something that happens to other people, even people who are, you know, have-have been physically active and whatever (P17)  Its isolated you massively. Um, I don’t know anybody else my age that goes through the pain I go through and so its finding people that understand that you’re a completely different person in some seasons, as to what you are in winter (P21)  I think definitely the sort of solidarity feeling that you’re not the only person experiencing something (P22)  I do think there’s people- you can become very ;lonely with chronic pain cause nobody can see it (P4)  I suppose, in a way, its sort of like, how can I put it? An AA for pain. You know, an alcoholics anonymous but for pain. I think they should have them- that might be a good idea (P5)  I think its important people can have someone to relate to in relation to their pain because it can feel quite isolating when they’re not able to do things they might have been able to do before, or that experience of feeling like they’re a burden, or that they shouldn’t be feeling the way they are (P6)  I find that there’s people that I’ve seen who have written forums on things I’ve read and stuff that will have like advice on there that I didn’t think of before (P10)  I suppose you can then say um, ‘what I find helpful is, you know, I was in a lot of pain but I forced myself to go for a walk. And do you know actually by the time I got back, I felt a lot better’, or that sort of thing (P17)  It will be good to hear other peoples ideas to see what, how they manage their certain pain because it might be something that I’ve never thought of before, It might be something that, you know, that’s not typically recommended but that you would see online through research (P6) |
| Opportunity | Physical | 1. Improved pain awareness and autonomy | This theme refers to the enhanced understanding of one’s pain patterns and triggers through tracking and self-monitoring via applications. By gaining better insight into when and why pain occurs, individuals can make more informed decisions about managing their condition. This increased awareness fosters a sense of autonomy, empowering individuals to take control of their pain management strategies and improve their overall health outcomes. | It might show a pattern. If they’ve not you know, not kept a diary or know their triggers for it. You know if it starts showing, like for me, I know that eating triggers it and the build-up of food over the day. So, if you didn’t know, something like that, you may look at it and think ‘oh why, why is it at this time, you know sort of everyday that its its at its peak level?’. Erm, I think it would be very helpful then (P18)  The occasions where a realisation did come up. You think like to yourself well. I know all this isn’t particularly useful. And then you check notes. And there are a few occasions where you think ‘oh, ok, yeah yeah that does link’. And then you think, ‘oh all right, well that’s something, you know that’s a step towards the right direction’. That’s, that’s good. It’s a new revelation I suppose, is probably the best, the best thing (P23)  I really like the idea of it being able to track your day-to-day things- that you wouldn’t think what have an- you know what I mean and spot patterns. And I thought that was really, that, that to me is like useful. I could understand because like that’s going to spot things that you cant spot, that’s really helpful. And to me, that’s kind of of one of the pain things, that I feel like I would use an app for (P24)  To then be able to see- cause I work with data, I like data. So if I could see if I’ve journaled everyday last month and I didn’t have any and then I didn’t do it for a few weeks and then they started creeping in, that would be really interesting for me to know. Because it would say to me maybe this is something that’s really helpful. So I think being able to log what preventative things you can do, is something that would be really useful, not just when you’re having an attack (P3)  Every month you usually feel around this, at this time of the month, and such like that. That would be really handy and like be interesting for me to see. So I’m working from another perspective (P20)  It was to have a visual reference so that, a doctor could look at it and see lots of different colours, the days I was suffering, bleeding- gynae bleeding- bone pain, muscle pain, diarrhoea (P14)  If I could maybe show like graph form as well, or sort of readable. Tangible information that when you go to your doctors appointment you could show them as well (P20)  Its hard cause it needs to be in some way measurable that can be communicated. So you an say ‘hey well, look you’re saying I need to experience this to have any clinical response to this pain, but I’m consistently here’ (P22)  I mean for some people tracking the frequency of it and their severity of it could be quite useful, I suppose and it might help them to build a better picture to go back for medical help to say; ‘look. I’ve been doing this and then here’s my results for the last two weeks. This is all the pain I’ve had. Please do something if you can’. That might be useful, but I think it just depends (P12) |
| Opportunity | Physical | 1. Accessibility | This theme refers to how pain self-management applications can be designed to be more inclusive and adaptable to a wide range of users, including those with physical or sensory disabilities. Accessibility improvements, such as voice recognition for users who may have difficulty using their hands or creating user-friendly interfaces for elderly individuals, are key to ensuring these apps are usable by a diverse population. Customization of the app to address specific pain types or needs further enhances its accessibility for all users. | I also have to remember that some people aren’t able to fill in with uh the hand, so you have to use voice recognition and, and obviously disability you know with deaf and blind people as well. It could be you know, it could be, adjusted for all sorts of people (P4)  Um, just little things like that, just to make it more accessible to everybody (P7)  I know that not everybody who suffers from chronic pain is elderly, that’s fine, but you know proportionally more people with chronic pain are elderly. So you need to, sort of, you need to um design something specifically for them, and I think its got to be as user friendly as possible and reassuring that they’re not going to break it (P2)  Yeah So having different sections of different types of pain and how to manage that type of pain. I think, I think would be better than just sort of an umbrella of here, here's how you can manage all pains. It's well, you know, that's probably not well suited to me. You know, it might be for some people, but not for me (P6) |

**S5.** *Researcher reflexivity*

I conducted all interviews as the lead researcher, a 27-year-old white female PhD candidate in Health Psychology, with a research focus on behaviour change and digital technology. I hold a BSc in Psychology, MSc Stage 1 Training in Health Psychology (Trainee Health Psychologist status) and have experience working in hospital settings. My training in Health Psychology has shaped my interest in biopsychosocial, holistic approaches to chronic condition management, including self-management and digital health interventions.

My interest in this topic is both professional and personal, I have a parent who lives with chronic pain, which has made me particularly sensitive to the complexities of managing long-term conditions. I also developed a strong interest in intervention design during prior academic work and am motivated to contribute to the national agenda to reduce opioid overuse through effective behavioural strategies.

The interview schedule was informed by the COM-B model and piloted with my supervisor, an experienced qualitative researcher and sport psychologist, to ensure clarity and relevance. Themes were later deductively mapped to the COM-B framework, which guided analysis without constraining theme development.

Throughout the study, I remained aware of how my background, assumptions, and personal motivations may have influenced my interactions with participants and interpretation of data. To support reflexivity, I maintained detailed field notes and participated in weekly reflective reviews with the research team, which included a chartered health psychologist experienced in behavioural interventions, a qualitative research expert, and a sport psychologist. These reviews supported a reflexive dialogue about our assumptions, individual experiences and potential biases.

We also used critical reflection strategies during analysis, such as “critical friends” discussions, and incorporated member reflections to ensure themes captured the lived experiences of chronic pain patients. These strategies helped us to continually consider how our individual perspectives might influence interpretation and allowed us to enhance scientific rigour of our research.
